# β_2_-adrenoceptor signaling reduction in dendritic cells is involved in the inflammatory response in adjuvant-induced arthritic rats

**DOI:** 10.1038/srep24548

**Published:** 2016-04-15

**Authors:** Huaxun Wu, Jingyu Chen, Shasha Song, Pingfan Yuan, Lihua Liu, Yunfang Zhang, Aiwu Zhou, Yan Chang, Lingling Zhang, Wei Wei

**Affiliations:** 1 Institute of Clinical Pharmacology of Anhui Medical University, Key Laboratory of Anti-Inflammatory and Immune Medicine, Ministry of Education, Anhui Collaborative Innovation Center of Anti-Inflammatory and Immune Medicine, Hefei, 230032, China

## Abstract

Rheumatoid arthritis (RA) is characterized by inflammation of the synovium, which leads to the progressive destruction of cartilage and bone. Adrenoreceptor (AR) signaling may play an important role in modulating dendritic cell (DC), which may be involved in the pathogenesis of RA. We examined the effect of the β-AR agonist isoprenaline (ISO) on DC function, the impact of the β_2_-AR agonist salbutamol on adjuvant-induced arthritic (AA) rats, and changes in β_2_-AR signaling in DCs during the course of AA. ISO inhibited the expression of the surface molecules CD86 and MHC-II, inhibited the stimulation of T lymphocyte proliferation by DC and TNF-α secretion, and promoted DC antigen uptake and IL-10 secretion. The effects of ISO on MHC-II expression, DC stimulation of T lymphocyte proliferation, and DC antigen uptake were mediated by β_2_**-**AR. Treatment with salbutamol ameliorated the severity of AA and histopathology of the joints and inhibited proliferation of thymus lymphocytes and FLS *in vivo.* β_2_-AR signaling was weaker in AA rats compared to the control. Elevated GRK2 and decreased β_2_-AR expression in DC cytomembranes were observed in AA and may have decreased the anti-inflammatory effect of β_2_-AR signaling. Decreased β_2_-AR signaling may be relevant to the exacerbation of arthritis inflammation.

Dendritic cells (DCs) are essential regulators of both the innate and acquired arms of the immune system. DCs likely contribute to the pathogenesis of autoimmune diseases such as rheumatoid arthritis (RA) in several ways^1^,^2^. Autoimmune models have revealed that DCs can prime MHC-restricted autoimmune responses in lymphoid organs^3^,^4^. Immature DCs efficiently capture antigens, including pathogens, particulates, and soluble foreign antigens or self-antigens^5^. Immature DCs express lower levels of maturation markers (CD80, CD86 and MHC-II) and produce little proinflammatory cytokines^6^. Synovial DCs exhibit upregulation of MHC and costimulatory molecules *in vivo*, suggesting activation. Both knockdown of costimulatory factors such as CD80 and CD86 and expression of immunosuppressive molecules in DCs have been exploited to generate tolerogenic DCs. These tolerogenic DCs effectively suppress the onset of collagen-induced arthritis, produce IL-10, and induce T-cell tolerance via immunosuppressive cytokines^7^. DCs must undergo a process of “maturation” involving upregulation of MHC, costimulatory molecules (CD80/86), activation markers and cytokine production to activate T cells. The DC maturation program can be stimulated by various mechanisms, including pathogen-derived molecules (lipopolysaccharide, DNA, RNA) and proinflammatory cytokines (TNF, IL-1, IL-6)^8^,^9^.

RA is characterized by inflammation of the synovium, which leads to progressive destruction of cartilage and bone^10^,^11^. Although the exact etiology of RA is unknown, there may be an interaction between the nervous system and inflammation in RA. The hypothalamic-pituitary-adrenal (HPA) axis and the sympathetic nervous system (SNS) release the neurotransmitters adrenaline (Adr) and norepinephrine (NE) and play an important role in RA^12^,^13^,^14^. Adr and NE subsequently activate adrenoreceptors on peripheral target tissues and regulate the corresponding physical effects. Adrenoreceptors (ARs) belong to the G protein-coupled receptor (GPCR) family, which is regulated by G protein-coupled receptor kinases (GRKs). There are three AR types (α1-, α2- and β-ARs), and each exhibits a different affinity for Adr and NE, depending on the receptor subtype and the tissue in which it is expressed^15^.

AR signaling may play an important role in modulating DC function during both the innate and adaptive immune responses^16^, and these changes in DC function may be involved in the pathogenesis of RA. Short-term exposure of murine bone marrow-derived dendritic cells (BMDCs) to NE reduces the release of IL-12 and stimulates the release of IL-10^17^,^18^. *In vitro*, NE reduces the ability of murine DCs to present antigen in a mixed lymphocyte reaction using an antigen-specific T cell clone^19^. Catecholamines may also inhibit the migration of DCs to the lymph nodes^20^.

What is the effect of β-ARs on the function of DCs? Is any such effect involved in the regulation of RA pathogenesis? In the present study, we investigated the effect of the β-AR agonist isoprenaline (ISO) on the function of DCs, the impact of the β_2_-AR agonist salbutamol on adjuvant-induced arthritic (AA) rats, and changes in β_2_-AR signaling in DCs from AA rats over the course of the disease. This research aims to elucidate the role of β_2_-AR signaling in RA pathogenesis and provide an experimental basis for the identification of new drug targets.

## Materials and Methods

### Animals

Male Sprague Dawley (SD) rats weighing 150–180 g were purchased from the Experimental Animal Center of Anhui Medical University (SPF, Certificate no. 2011–002). The animals were housed in a room with a controlled ambient temperature (22 ± 2 °C) and humidity (50% ± 10%), with food and water ad libitum. All procedures were performed in accordance with the guidelines of the Animal Care and Use Committee of Anhui Medical University and were approved by the Ethics Committee of Anhui Medical University.

### Reagents

Recombinant rat interleukin 4 (IL-4) and granulocyte-macrophage colony-stimulating factor (GM-CSF) were purchased from Peprotech (Rocky Hill, NJ, USA). RPMI-1640 medium, Dulbecco’s modified Eagle’s medium (DMEM) and fetal bovine serum (FBS) were from Hyclone (Logan, UT, USA). Phycoerythrin (PE)-conjugated anti-CD80, -CD86, and -MHC-II, Alexa Fluor 647-CD103 and isotype control antibody were purchased from BioLegend (San Diego, CA, USA). The antibodies against β_2_-AR, GRK2 and β-actin were from Abcam (Cambridge, UK). Horseradish peroxidase (HRP)-labeled goat anti-rabbit and goat anti-mouse antibodies were acquired from Santa Cruz Biotechnology (CA, USA). LPS, FITC-dextran (40 kD), isoprenaline hydrochloride, CGP20712A, ICI118551 and CCK-8 were purchased from Sigma (St. Louis, MO, USA). Salbutamol sulfate was from Shanghai Xudong Haipu Pharmaceutical Co., Ltd. (Shanghai, China). ELISA kits for interleukin 10 (IL-10) and TNF-α were purchased from RayBiotech, Inc. (Norcross, GA, USA).

### Preparation of BMDCs

Bone marrow cells were collected from the tibias and femurs of SD rats by flushing the bones. The cells were pipetted vigorously up and down several times to obtain single-cell suspensions and passed through a nylon cell strainer to remove small pieces of bone and debris. The cells were cultured in RPMI-1640 medium containing 10% FBS at a density of 5 × 10^6^ cells/ml in 6-well plates. Three hours later, non-adherent cells were discarded, and new medium supplemented with IL-4 (10 ng/ml) and GM-CSF (10 ng/ml) was added. The cultures were fed fresh medium and cytokines every 3 days. On day 5, isoprenaline (10^−5^, 10^−6^, or 10^−7^ mol/l) was added; no isoprenaline was added to the control group. Simultaneously, LPS (100 ng/ml) was added to the BMDCs, except the LPS(−) group. Loosely adherent clusters were harvested on day 6–8 and used for experiments.

### Phenotyping of DCs

The BMDCs (1 × 10^6^ cells) prepared above and spleen lymphocytes were acquired for each sample and stained for CD103 (Alexa Fluor 647), CD80 (PE), CD86 (PE), MHC-II (PE), or the corresponding isotype control for 30 min at 37 °C. Because CD103 is a specific marker for rat DCs, CD103^+^ cells were gated; within this population, the expression of CD80, CD86 and MHC-II on DCs was measured by flow cytometry. Data analysis was performed using FlowJo analysis software (Tree Star, Ashland, OR, USA) and reported as the mean fluorescence intensity (MFI).

### Quantification of antigen uptake by BMDCs

The BMDCs prepared above were incubated in complete medium with FITC-dextran at a final concentration of 1 mg/ml at 37 °C for 2 h. Background staining at 4 °C was used as a negative control. The BMDCs were washed three times with cold phosphate-buffered saline (PBS), and the incorporation of FITC-dextran was analyzed by flow cytometry. The data are presented as mean fluorescence intensities (MFIs).

### Mixed lymphocyte reaction (MLR)

The BMDCs prepared above were harvested on day 7 and treated with mitomycin (25 μg/ml) at 37 °C for 30 min, then washed twice with PBS. Rat splenic T lymphocytes (2 × 10^5^ cells/well) were collected through nylon wool and co-cultured with these BMDCs in 96-well plates at ratios of 10:1 for 48 h at 37 °C. Four hours before the end of the incubation, 20 μl of CCK-8 was added to each well, and the absorbance at 490 nm was determined using a multi-well plate reader (Beckman, USA). The experiments were conducted in triplicate for each condition.

### Determination of cytokines IL-10 and TNF-α in BMDC supernatants

The supernatants were collected on day 7 of BMDC culture, and IL-10 and TNF-α levels were immediately assayed using commercial test kits according to the manufacturer’s protocols. The kit enables the quantitative measurement of rat IL-10 and TNF-α in serum, plasma, and cell culture supernatants. The absorbance at 405 nm was measured using a Multiskan Spectrum. Each sample was assayed in duplicate.

### Induction and treatment of AA rats

The rat AA model was induced by a single intradermal injection of 0.1 ml of complete Freund’s adjuvant (CFA) into the right hind footpad. The day of CFA injection was designated day 0, and the secondary inflammatory reaction occurred after day 14 (d14).

After the onset of arthritis on d14, the animals were randomly allocated to 6 groups: control, AA model, salbutamol (0.75, 1.5, 3.0 mg/kg, intragastric administration, for 14 days), and MTX (0.5 mg/kg, intragastric administration, every three days, for 5 times). The rats received medication from d15 to d28. The rats in the normal control and AA model groups received an equal volume of 0.5% sodium carboxymethylcellulose (CMC-Na) at the same time points.

### Evaluation of arthritis

To evaluate the severity of arthritis, the secondary inflammatory paw (left hind) swelling of rats was evaluated at 0, 7, 14, 21, 28, 35 days using a Paw Volume Meter^21^: paw swelling degree = paw swelling (d7, 14, 21, 28, 35) - paw swelling (d0).

### Histopathological examination and evaluation

Rats were sacrificed on day 28 to dissect the left hind knee joint. The joints were removed, fixed in formalin, decalcified in 10% ethylenediaminetetraacetic acid (EDTA) and embedded in paraffin for histopathological analysis. Serial paraffin sections were stained with hematoxylin and eosin (H&E).

The severity of arthritis in the joint was graded from 0 to 4 according to the intensity of lining layer hyperplasia, mononuclear cell infiltration and pannus formation, as described previously (0 = normal ankle joint, 1 = normal synovium with occasional mononuclear cells, 2 = definite arthritis with a few layers of flat to rounded synovial lining cells and scattered mononuclear cells and dense infiltration with mononuclear cells, 3 = clear hyperplasia of the synovium with three or more layers of loosely arranged lining cells and dense infiltration with mononuclear cells, 4 = severe synovitis with pannus and erosion of articular cartilage and subchondral bone)^22^.

### Assay of thymus and spleen lymphocyte proliferation

Rats were sacrificed on d35 after immunization. The thymus and spleen were dislodged under sterile conditions. The cells were suspended in a lymphocyte separation medium and washed three times with PBS. Thymus cells (200 μl; 1 × 10^6^) from each group were placed in 96-well plates with ConA (5 mg/l), and spleen cells (200 μl; 1 × 10^6^) from each group were placed in 96-well plates with LPS (4 mg/l); all suspensions were prepared in triplicate and incubated at 37 °C in 5% CO_2_ for 48 h. Four hours before the end of the incubation, 20 μl of CCK-8 was added to each well. The absorbance was measured by a Multiskan Spectrum (BioTek Co., Ltd, USA). The results are presented as the average of triplicate counts.

### Culture and proliferation assay of fibroblast-like synoviocytes (FLSs)

The rats were anesthetized and sacrificed on d35 after immunization, and the synovial tissues from the knees joints were excised. FLSs were isolated from individual tissues using a tissue transplantation method and cultured in DMEM supplemented with 20% fetal calf serum, penicillin (200 U/ml), and streptomycin (200 ng/ml) at 37 °C in 5% CO_2_. Confluent adherent cells were trypsinized, split in a 1:3 ratio, and re-cultured in medium. The spindle-shaped cells obtained from passages 3 to 5 consisted of a homogeneous population of synoviocytes. The cells were resuspended at a cellular density of 1.0 × 10^5^ cells/ml in 96-well flat-bottomed culture plates. The cultures were incubated at 37 °C in 5% CO_2_ for 48 h. Four hours before the end of the incubation, 20 μl of CCK-8 was added to each well. The absorbance was measured by a Multiskan Spectrum (BioTek Co., Ltd, USA). The results are presented as the average of triplicate counts.

### Western blot analysis

Rats were sacrificed on d0, d7, d14, d21, and d28 after immunization. BMDCs were isolated from each group and prepared as above, then lysed in cell lysis buffer with 1 mM PMSF, followed by centrifugation (100,000 rpm) for 60 min; the precipitates were diluted to 4 mg protein/ml and stored frozen at −80 °C until use. The precipitate mainly comprised cytomembrane proteins. A total of 50 μg of denatured protein was separated by 10% SDS-PAGE, transferred onto polyvinylidene fluoride membranes (PVDF membranes, Millipore, USA), and then incubated with primary antibodies to β_2_-AR and GRK2 (1:1000) and mouse monoclonal anti-β-actin (1:500) at 4 °C overnight. Then, the membranes were incubated with secondary antibodies conjugated to HRP, and detection was achieved by measuring the chemiluminescence of the blotting agent after exposure of the filters to films. Finally, the densities of the bands were quantified with a computerized densitometer (ImageJ Launcher, Broken Symmetry Software). Equivalent protein loading and transfer efficiency were verified by staining for β-actin.

### Statistical analysis

Data are expressed as the mean and standard deviation (SD). Analysis of variance (ANOVA) and Student’s t-test were performed to determine significant differences between groups. Calculations were performed using the SPSS version 11.5 statistical package. Values of P < 0.05 were considered significant.

## Results

### ISO inhibits BMDC maturation

**Figure 1:**
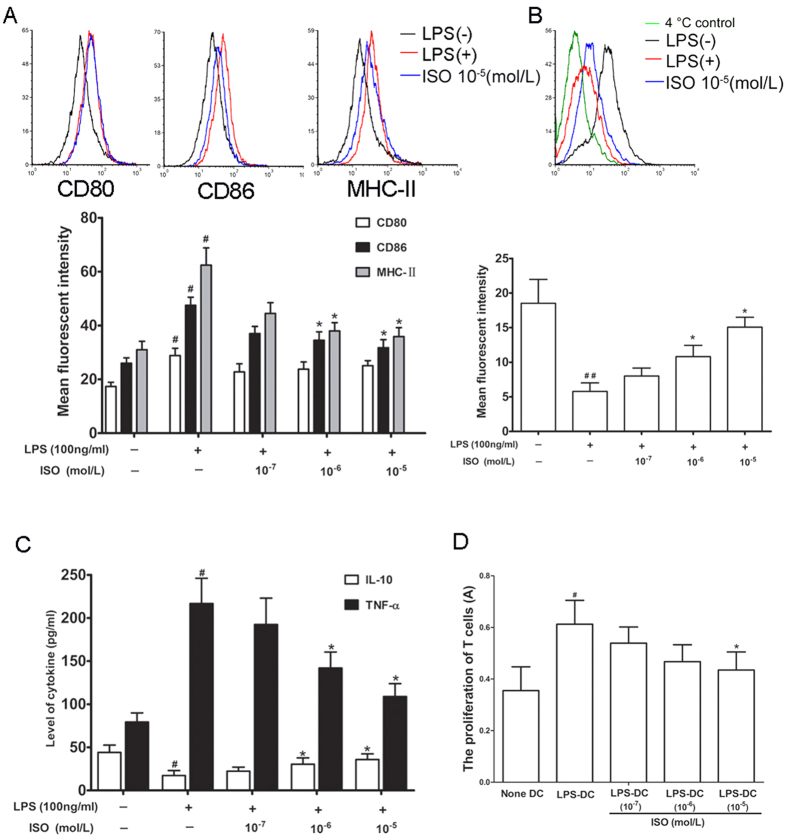
The effects of ISO on BMDCs. (**A**) The effect of ISO on the phenotype of BMDCs. CD103^+^ cells were gated, and within this population, the expression of CD80, CD86 and MHC-II on DCs was measured as the mean fluorescence intensity (MFI). Data are shown as the mean ± SD of 3 replicate experiments. ^#^P < 0.05, compared with LPS(−) group, *P < 0.05, compared with LPS(+) control group. (**B**) The effect of ISO on the antigen uptake capability of BMDCs. The incorporation of FITC-dextran was analyzed by MFI. Data are shown as the mean ± SD of 3 replicate experiments. ^#^P < 0.05, compared with LPS(−) group, *P < 0.05, compared with LPS(+) control group. (**C**) The effect of ISO on IL-10 and TNF-α levels in BMDCs. Data are shown as the mean ± SD of 3 replicate experiments. ^#^P < 0.05, compared with LPS(−) group, *P < 0.05, compared with LPS(+) control group. (**D**) The effect of ISO on MLR of BMDCs. Data are shown as the mean ± SD of 3 replicate experiments. ^#^P < 0.05, compared with (None DC) group, *P < 0.05, compared with (LPS-DC) control group.

ISO (10^−5^, 10^−6^, or 10^−7^ mol/L) and LPS (100 ng/ml) were added to BMDCs (except the LPS(−) control group), followed by staining (1 × 10^6^ cells). Compared with the LPS(−) group, LPS significantly promoted the expression of the surface molecules CD80, CD86 and MHC-II. ISO (10^−5^ and 10^−6^ mol/l) significantly inhibited the expression of the surface molecules CD86 and MHC-II on DCs treated with LPS but had no significant effect on the expression of CD80 (Fig. 1A).

ISO (10^−5^, 10^−6^, or 10^−7^ mol/l) and LPS (100 ng/ml) were added to BMDCs (except the LPS(−) group), and FITC-dextran was then used as an antigen to evaluate the antigen uptake capability of BMDCs by flow cytometry. Background staining at 4 °C was used as a negative control. As shown in a representative experiment (Fig. 1B), endocytosis was easily observed based on the MFI. Compared with the LPS(−) group, LPS significantly inhibited the antigen uptake capability of BMDCs, whereas ISO (10^−5^ and 10^−6^ mol/l) significantly upregulated the antigen uptake capability of BMDCs treated with LPS.

Supernatants were collected from BMDCs treated with ISO (10^−5^, 10^−6^, or 10^−7^ mol/l) and LPS, and the levels of IL-10 and TNF-α were measured by ELISA. ISO (10^−5^ and 10^−6^ mol/l) significantly promoted IL-10 secretion and inhibited TNF-α secretion from the BMDCs treated with LPS (Fig. 1C).

DCs play pivotal roles in T-cell-mediated immune responses, and thus we also investigated the ability of DCs to activate T cells using MLRs. T cells and BMDCs treated with ISO (10^−5^, 10^−6^, or 10^−7^ mol/l) and LPS were mixed, and their ability to induce allogeneic T-cell proliferation was evaluated. ISO (10^−5^ mol/l) inhibited the stimulation of T lymphocyte proliferation by LPS treatment of BMDCs (Fig. 1D).

### ISO reduces MHC-II expression and the mixed lymphocyte reaction in BMDCs and promotes antigen uptake function mainly mediated by β_2_-AR signaling

ISO inhibited the expression of the BMDC surface molecules CD86 and MHC**-**II, significantly promoted antigen uptake capability, and inhibited the stimulation of T lymphocyte proliferation. To further explore the β-AR subtypes mediating these functions, a selective β_1_**-**AR antagonist (CGP20712A) and a β_2_-AR antagonist (ICI118551) were used to identify the role of β**-**AR subtypes in BMDCs treated with LPS.

**Figure 2:**
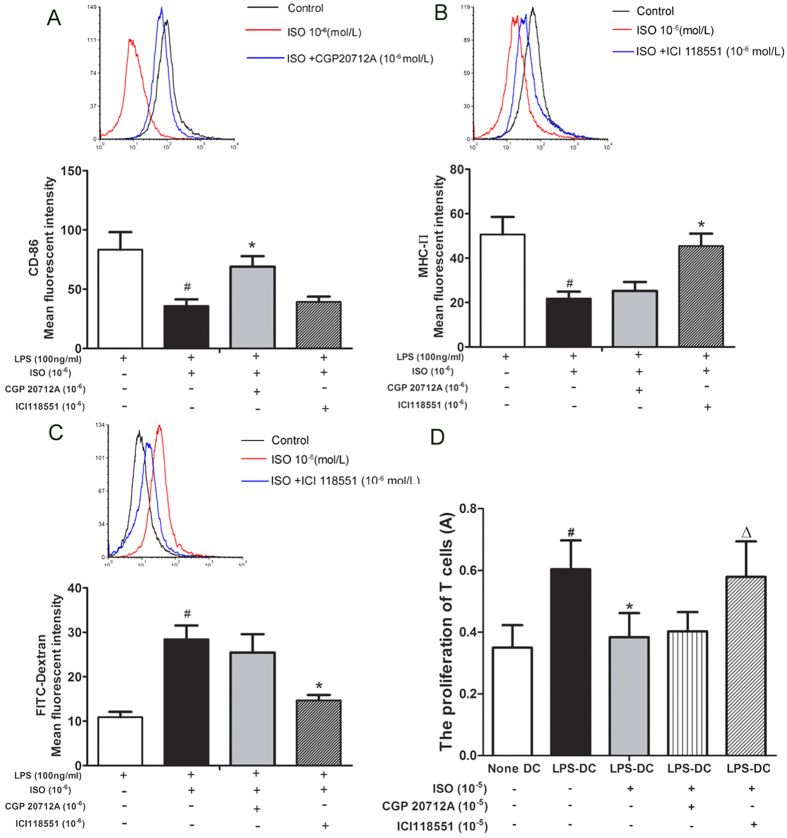
The effects of selective β-AR antagonists on BMDCs stimulated with ISO. (**A**) The effect of selective β-AR antagonists on CD86 on BMDCs stimulated with ISO. CD103^+^ cells were gated, and within this population, the expression of CD86 on DCs was measured. Data are shown as the mean ± SD of 3 replicate experiments. ^#^P < 0.05, compared with LPS(+) control group, *P < 0.05, compared with ISO (10^−6^ mol/l). (**B**) The effect of selective β-AR antagonists on MHC-II on BMDCs stimulated with ISO. CD103^+^ cells were gated, and within this population, the expression of MHC-II on DCs was measured. Data are shown as the mean ± SD of 3 replicate experiments. ^#^P < 0.05, compared with LPS(+) control group, *P < 0.05, compared with ISO (10^−6^ mol/l). (**C**) The effect of selective β-AR antagonists on antigen uptake capability in BMDCs stimulated with ISO. Data are shown as the mean ± SD of 3 replicate experiments. ^#^P < 0.05, compared with LPS(+) control group, *P < 0.05, compared with ISO (10^−6^ mol/l). (**D**) The effect of selective β-AR antagonists on MLR with BMDCs. Data are shown as the mean ± SD of 6 replicate experiments. ^#^P < 0.05, compared with (None DC) group, *P < 0.05, compared with (LPS-DC) group, ^∆^P < 0.05, compared with ISO (10^−5^ mol/l).

On BMDCs treated with LPS, ISO (10^−6^ mol/l) significantly decreased CD86 expression, and the selective β_1_**-**AR antagonist CGP20712A (10^−6^ mol/l) antagonized the effect of ISO, whereas the selective β_2_**-**AR antagonist ICI 118551 (10^−6^ mol/l) had no obvious effect (Fig. 2A).

ISO (10^−6^ mol/l) significantly decreased MHC**-**II expression on BMDCs compared with the control group treated with LPS, and the selective β_2_**-**AR antagonist ICI 118551 (10^−6^ mol/l) antagonized the effect of ISO, whereas the selective β_1_**-**AR antagonist CGP20712A (10^−6^ mol/l) had no obvious effect (Fig. 2B).

In BMDCs treated with LPS, ISO (10^−6^ mol/l) significantly promoted antigen uptake capability. The selective β_2_**-**AR antagonist ICI 118551 (10^−6^ mol/l) antagonized the effect of ISO, whereas the selective β_1_**-**AR antagonist CGP20712A (10^−6^ mol/l) had no obvious effect (Fig. 2C).

ISO (10^−5^ mol/l) inhibited the stimulation of T lymphocyte proliferation in BMDCs treated with LPS. The selective β_2_**-**AR antagonist ICI 118551 (10^−6^ mol/l) antagonized the effect of ISO, whereas the selective β_1_**-**AR antagonist CGP20712A (10^−6^ mol/l) had no obvious effect (Fig. 2D).

The above results demonstrate that the effects of ISO on MHC-II expression, mixed lymphocyte reactions with BMDCs, and antigen uptake function are mediated by β_2_**-**ARs on BMDCs treated with LPS.

### The β_2_-AR agonist salbutamol attenuates the inflammatory response in adjuvant-induced arthritic rats

Because β_2_-AR activation affected the function of BMDCs, we next examined the effect of the β_2_-AR agonist salbutamol on adjuvant-induced arthritis mediated by DCs.

**Figure 3:**
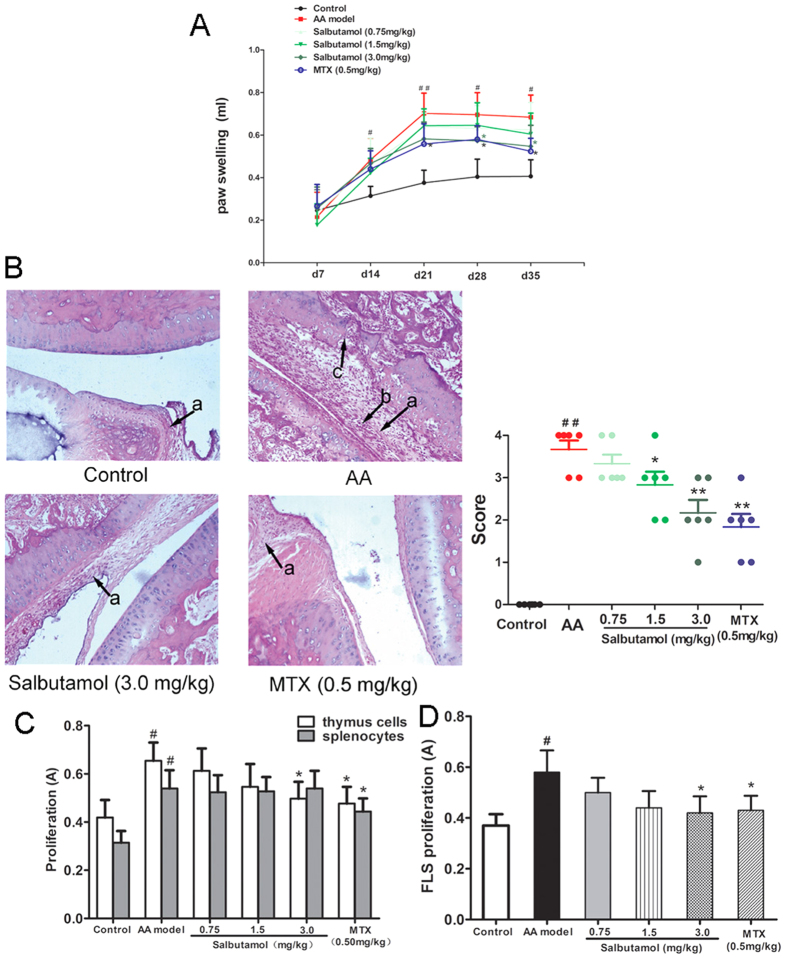
The effects of salbutamol on general indicators in AA rats. (**A**) The effects of salbutamol on degree of paw swelling in AA rats. SD rats were immunized with CFA on day 0 and then administered salbutamol or MTX for 14 days, from d15 to d28. The paw swelling degree of AA rats was evaluated on d0, d7, d14, d21, d28, and d35. Data are expressed as the mean ± SD for 6 animals in each group. ^##^P < 0.01 *vs* Normal Control; *P < 0.05 *vs* AA group. (**B**) The effects of salbutamol on the joint histopathology of AA rats. Arrows a represent synovial hyperplasia, arrows b represent inflammatory cell infiltration, and arrows c represent pannus formation and bone erosion. Data are expressed as the mean** **±** **SD for 6 animals in each group. ^##^P < 0.01 *vs* Normal control; *P < 0.05, **P < 0.01 *vs* AA group. (**C**) The effects of salbutamol on the proliferation of thymus and spleen lymphocytes. Data are expressed as the mean ± SD for 6 animals in each group. ^#^P < 0.05 compared with control; *P < 0.05 compared with AA group. (**D**) The effects of salbutamol on the proliferation of FLSs. Data are expressed as the mean ± SD for 6 animals in each group. ^#^P < 0.05 compared with control; *P < 0.05 compared with AA group.

After the onset of arthritis on day 14, there was a significant increase in paw swelling degree in AA rats compared with the control. Salbutamol (3.0 mg/kg) clearly attenuated the degree of paw swelling 14 days after administration (on d28), compared with the AA model (Fig. 3A).

The ameliorating effect of salbutamol on AA was further confirmed by histopathological analysis of joints. Synoviocytes were in a monolayer, and there was no infiltration of inflammatory cells in the control rat knee joints. In the AA model group, synoviocytes proliferated over three layers with pannus formation, and articular cartilage and bone were eroded and infiltrated with inflammatory cells. Histopathological evaluation revealed that synovial hyperplasia, cell infiltration, pannus and bone erosion were significantly increased compared with normal controls. These abnormalities were significantly alleviated in AA rats after administration of salbutamol (1.5, 3.0 mg/kg) (Fig. 3B).

Proliferation was significantly increased in thymus lymphocytes, spleen lymphocytes and FLSs from AA rats compared with control rats. Treatment with salbutamol (3.0 mg/kg) significantly inhibited thymus lymphocyte and FLS proliferation compared with cells from AA rats, with no obvious effect on the proliferation of spleen lymphocytes (Fig. 3C,D).

**Figure 4:**
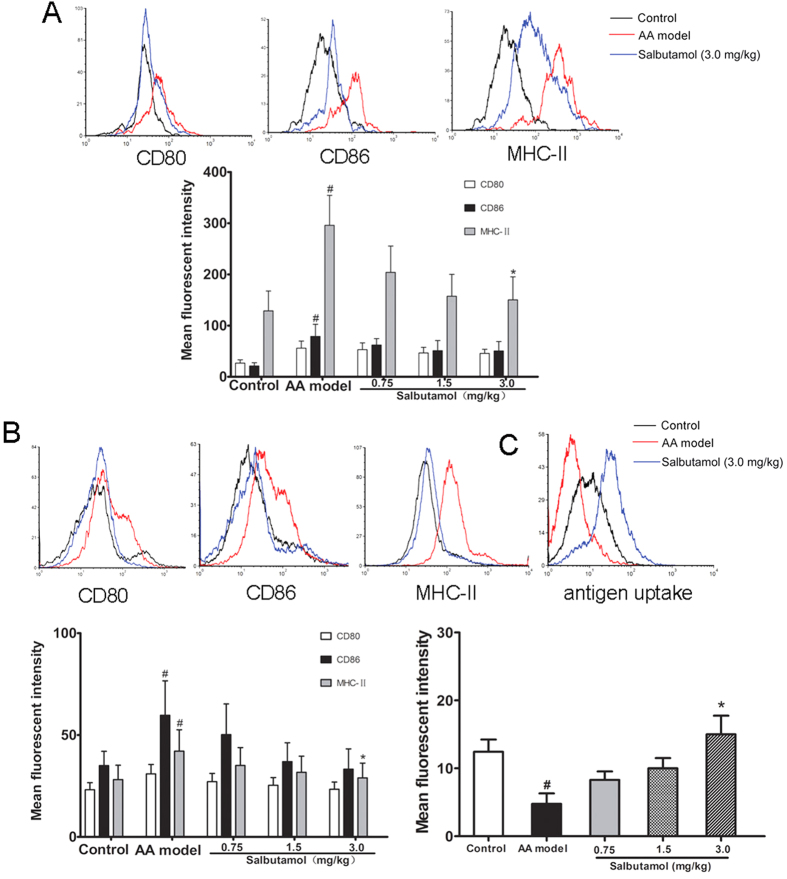
The effects of salbutamol on DCs from spleen lymphocytes and BMDCs in AA rats. (**A**) The effects of salbutamol on the phenotype of DCs from spleen lymphocytes in AA rats. CD103^+^ cells were gated, and within this population, the expression of CD80, CD86 and MHC-II on DCs was measured by the mean fluorescence intensity. (**B**) The effects of salbutamol on the phenotype of BMDCs from AA rats. CD103^+^ cells were gated, and within this population, the expression of CD80, CD86 and MHC-II on DCs was measured by the mean fluorescence intensity. (**C**) The effects of salbutamol on the antigen uptake capability of BMDCs from AA rats. Data are shown as the mean ± SD for 3 animals in each group. ^#^P < 0.05 compared with control; *P < 0.05 compared with AA model.

The expression of the surface molecules CD86 and MHC-II on DCs on spleen lymphocytes was significantly increased in AA rats compared with the control rats, and treatment with salbutamol (3.0 mg/kg) significantly inhibited the expression of MHC-II compared with AA rats, with no obvious effect on the expression of CD80 and CD86 (Fig. 4A).

The expression of the surface molecules CD86 and MHC-II was significantly increased on BMDCs from AA rats, and treatment with salbutamol (3.0 mg/kg) significantly inhibited the expression of MHC-II and promoted antigen uptake capability compared with AA rats, with no obvious effect on the expression of CD80 and CD86, consistent with the results for DCs from spleen lymphocytes (Fig. 4B,C).

### β_2_-AR signaling is weaker in BMDCs from AA rats than in those from control rats

**Figure 5:**
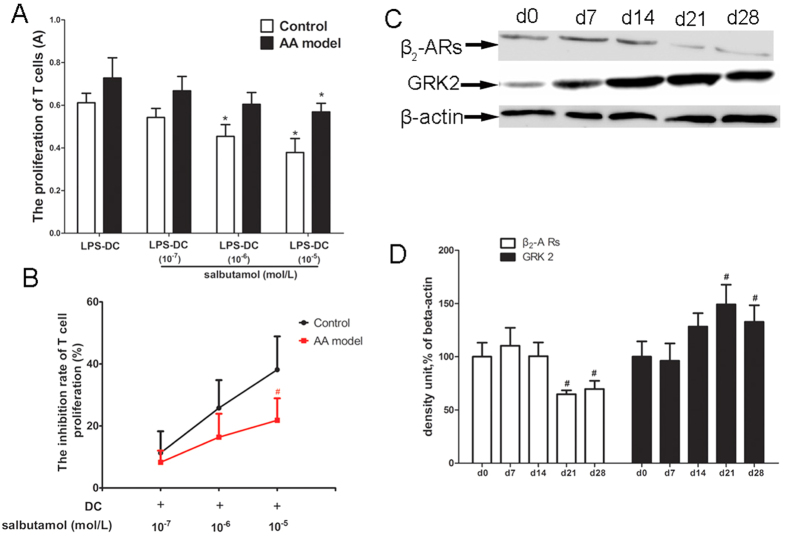
β_2_-AR signaling is weaker in BMDCs from AA rats than in BMDCs from control rats. (**A**) The effect of salbutamol on MLR of BMDCs from control and AA rats. BMDCs generated from control (non AA) and AA rats were treated with salbutamol (10^−5^, 10^−6^, or 10^−7^ mol/l) *ex vivo*. Proliferation was measured by CCK-8 assay. *P < 0.05, compared with (LPS-DC) group. (**B**) T cell proliferation inhibition rate were analyzed. Data are shown as the mean ± SD for 6 animals in the AA and control groups, ^#^P < 0.05, compared with control group. (**C**) The expression of β_2_-AR and GRK2 in BMDCs collected on d0, d7, d14, d21, and d28 from AA rats. (**D**) Densitometric analysis of the above immunoblots in bar chart form. Data are expressed as the mean ± SD for 3 animals in each group. ^#^P < 0.05 *vs* d0 control.

BMDCs generated from control (non AA) and AA rats were treated with salbutamol (10^−5^, 10^−6^, or 10^−7^ mol/l) *ex vivo.* T cells were then mixed, and the ability to induce allogeneic T cell proliferation was assessed. Salbutamol (10^−5^ or 10^−6^ mol/l) significantly inhibited MLR of BMDCs from control rats, and only the highest concentration of salbutamol (10^−5^ mol/l) significantly inhibited MLR of BMDCs from AA rats. Salbutamol inhibited the stimulation of T lymphocyte proliferation in control and AA rats, but the inhibitory effect of salbutamol (10^−5^ mol/l) was stronger for cells from the control group compared to cells from the AA group. These results suggest that β_2_-AR signaling may be weaker in BMDCs from the AA model (Fig. 5A,B).

To further explore β_2_-AR signaling changes in BMDCs from AA rats, we investigated the expression of β_2_-AR and GRK2 in the cytomembrane in different disease stages of AA. The expression of β_2_-AR significantly decreased on d21 and d28, whereas GRK2 significantly increased on d21 and d28, consistent with peak disease (Fig. 5C,D).

## Discussion

DCs are potent antigen-presenting cells (APCs) that play a major role in the regulation of immune responses to a variety of antigens. DCs first capture antigens via endocytosis and then present these antigens in the context of MHC-II molecules at the cell surface to activate antigen-specific CD4^+^ T cells. Immature DCs have a strong ability for antigen uptake but express low levels of the costimulatory molecules CD80/86 and MHC-II and weakly stimulate T-cell proliferation, characteristics of tolerogenic DCs^23^,^24^. Therefore, the first focus of our study was to observe the phenotypic and functional changes in DCs after treatment with ISO. CD103 is a specific marker for rat DCs and is expressed variably on DCs generated *in vitro*^25^. To minimize interference from other cells, we chose to gate on CD103^+^ cells. We observed that CD86 and MHC-II expression on BMDCs decrease following ISO treatment. Further study revealed that ISO promotes antigen uptake capability and IL-10 secretion, decreases the TNF-α levels, and inhibits the stimulation of T-lymphocyte proliferation. Thus, ISO inhibits DC maturation and promotes tolerance. To further explore the β-AR subtypes mediating these functions, a selective β_1_-AR antagonist (CGP20712A) and β_2_-AR antagonist (ICI118551) were used. The results demonstrated that the effects of ISO on MHC-II expression, mixed lymphocyte reactions of BMDCs, and antigen uptake function were mediated by β_2_-AR.

In the present study, we demonstrated that ISO promotes the antigen uptake capability of DCs via β_2_-AR signaling. Our results are consistent with studies have demonstrated that noradrenaline enhances DC antigen uptake^26^. β-AR stimulation inhibited the expression of the costimulatory molecule CD86, MHC-II and TNF-α and promoted IL-10 secretion. Previous research indicates that β-AR stimulation may inhibit the translocation of the transcription factor NF-κB to the nucleus. NF-κB in DCs is essential for upregulating the expression of CD86, MHC-II, and immunostimulatory cytokines such as IL-12 and TNF-α^27^,^28^. We infer that inhibiting the nuclear translocation of NF-κB by β-AR stimulation may explain the observed effects on DC function.

Considering the effect of β_2_-AR signaling on MHC-II expression, the BMDC MLR results, and antigen uptake function, we further investigated the effect of the β_2_-AR selective agonist salbutamol on the inflammatory response of AA rats *in vivo*. We evaluated disease progression based on the degree of paw swelling degree and joint histopathology in AA rats. Treatment with salbutamol significantly ameliorated the severity of arthritis and abnormal joint histopathology. A related β_2_-AR agonist, terbutaline, produced similar results in collagen-induced arthritis and AA^29^,^30^ in research focused on T and B lymphocytes. Despite the known inhibitory effects of β_2_-AR agonists on arthritis, Straub *et al*. observed that splenic IFN secretion is stimulated by NE via β-ARs, contributing to a proinflammatory effect after the onset of CIA. The reason for this effect is not clear but could be related to disease progression^31^. Moreover, salbutamol significantly inhibited thymus lymphocyte and FLS proliferation in AA rats *in vivo.* This finding is consistent with the known effects of β_2_-AR agonists on T lymphocyte proliferation and differentiation^30^. The failure to observe an effect on the proliferation of spleen lymphocytes is inconsistent with reports that the β_2_-AR agonist terbutaline inhibits the proinflammatory effects of IL-7R^+^ B cells^29^. This discrepancy may be attributable to differences in the reactivity of total splenocytes to β_2_-AR agonists compared with IL-7R^+^ B cells. How does the β_2_-AR selective agonist salbutamol play a therapeutic role in AA rats? Salbutamol significantly inhibited the expression of MHC-II in DCs from splenocytes of AA rats, and MHC-II is an important surface molecule in the activation of Ag-specific CD4^+^ T cells. Synovial fluid DCs in RA are more mature than normal: they express high levels of MHC-II molecules and potently stimulate a variety of T cell responses^32^,^33^. Therefore, inhibiting the expression and activity of MHC-II reduces the inflammatory reaction and promotes immune tolerance. Similar effects of salbutamol were observed in BMDCs from AA rats, including inhibition of the expression of MHC-II and promotion of antigen uptake capability compared with AA rats. The above results demonstrate that the β_2_-AR selective agonist salbutamol can ameliorate the severity of arthritis, perhaps by inhibiting the maturation of DCs and promoting their tolerance. This conclusion is consistent with a report by Cobelens and coworkers that salbutamol potentiates oral induction of tolerance, suppressing adjuvant arthritis and antigen-specific immunity^34^. Our findings may explain, in part, the mechanism underlying the induction of this tolerance.

Because β_2_-AR signaling activation can inhibit DC maturation and promote tolerance, we examined the potential differences in the effect of β_2_-AR signaling activation on DCs in normal and AA rats? The effects of ISO on BMDC MLRs were mediated by β_2_-AR. BMDCs from control and AA rats were treated with salbutamol and mixed with T cells, and the ability to induce allogeneic T-cell proliferation was compared. The inhibitory effect of salbutamol on the AA group was weaker than that on the control group. These suggest that β_2_-AR signaling may be weaker in BMDCs from the AA rat model. To further confirm β_2_-AR expression in DCs at different stages of AA, we detected the expression of β_2_-ARs and the negative regulatory protein GRK2 in the cytomembrane of DCs. β_2_-AR expression significantly decreased at d21 and d28, whereas GRK2 expression significantly increased at d21 and d28, consistent with peak disease. Reduction of β_2_-AR signaling may be responsible, at least in part, for DC dysfunction in AA.

Under normal physiological conditions, agonist stimulation by β_2_-AR induces the activation of the Gs/cAMP/protein kinase A (PKA) pathway. Agonist stimulation triggers β_2_-AR desensitization involving GRK phosphorylation, which in turn triggers arrestin binding, internalization, recycling and resensitization^35^,^36^. In the AA model, one of the hallmark molecular abnormalities is elevation of GRK2, which is observed in synovial tissues and draining lymph nodes in experimental arthritis^37^,^38^. Consequently, GRK2 may be an important molecular target in arthritis. The elevation of GRK2 in DC cytomembranes decreased the expression of β_2_-AR, which diminished the anti-inflammatory effect of β_2_-AR signaling. β_2_-AR expression significantly decreased at the peak of disease, which may be relevant to inflammatory response exacerbation.

## Conclusions

In summary, the present study reports that the effects of ISO on MHC-II expression, mixed lymphocyte reactions, and the antigen uptake function of BMDCs are mediated by β_2_-AR signaling. Treatment with the β_2_-AR selective agonist salbutamol significantly ameliorated the severity of arthritis and abnormal histopathology of joints and inhibit thymus lymphocyte and FLS proliferation from AA rats *in vivo.* These effects may be mediated by inhibition of DC maturation and promotion of tolerance. However, elevated GRK2 and decreased β_2_-AR in DC cytomembranes were observed in the AA model and may have decreased the anti-inflammatory effect of β_2_-AR signaling. Abnormal β_2_-AR signaling may be relevant to the exacerbation of arthritic inflammation. GRK2 is an important molecule that induces a decrease in β_2_-AR; therefore, drug targeting of GRK2 may be a direction for future research on RA.

## Additional Information

**How to cite this article**: Wu, H. *et al*. β_2_-adrenoceptor signaling reduction in dendritic cells is involved in the inflammatory response in adjuvant-induced arthritic rats. *Sci. Rep.* **6**, 24548; doi: 10.1038/srep24548 (2016).
